# Oral health and dental caries experience among students aged 7–15 years old with autism spectrum disorders in Tehran, Iran

**DOI:** 10.1186/s12887-022-03178-5

**Published:** 2022-03-05

**Authors:** Hedieh Piraneh, Mahdia Gholami, Katayoun Sargeran, Ahmad Reza Shamshiri

**Affiliations:** 1 Department of Community Oral Health, Research Center for Caries Prevention, Dentistry Research Institute, School of Dentistry, Tehran University of Medical Sciences, North Kargar Ave, 1439955991 Tehran, Iran; 2 Department of Community Oral Health, School of Dentistry, Tehran University of Medical Sciences, North Kargar Ave, 1439955991 Tehran, Iran

**Keywords:** Oral health, Autistic disorder, Dental caries, Oral hygiene

## Abstract

**Background:**

Autism spectrum disorders (ASD) are a set of developmental, psychological, and neurological disorders that occur in early childhood. The most important characteristic of individuals with autism is difficulty in social interactions and communication. Researchers in the field of oral health have not paid enough attention to these individuals due to their specific behavioral characteristics. Therefore, due to the limitations of the studies in this field in Iran, increasing prevalence of autism, and importance of oral health in people with ASD, this study was conducted to evaluate the oral health status of primary school students with autism (7–15 years old) in autism schools in Tehran.

**Methods:**

Students from seven governmental special primary schools in Tehran were selected for this study. Data about oral health behavior and the presence of each of the seven barriers of tooth brushing task was collected via questionnaires completed by parents. During the dental examination, the cooperation level according to the Frankle Index, oral hygiene status according to the Simplified Oral Hygiene Index, and caries experience (DMFT) of the students were recorded by a calibrated dentist. A psychologist assessed the level of ASD using the Diagnostic and Statistical Manual of Mental Disorders, 5th Edition (DSM5). Data analysis including descriptive and regression analysis was done using the SPSS software version 24.

**Results:**

Two hundred and seventeen students aged 7–15 years participated in this study, of whom 65.4% brushed their teeth once or more every day, 85.7% had sugary snacks twice or less per day, 73.7% used fluoridated tooth paste, and 80% brushed their teeth with parents’ help. The most common barrier to tooth brushing was difficulty in brushing (51.6%). The cooperation level was definitely positive in 46.1%. The mean Simplified Oral Hygiene Index (OHI-S) and caries experience (DMFT) scores were 1.92 ± 0.55 and 2.36 ± 2.38, respectively.

**Conclusion:**

The clinical indices of OHI-S and caries experience (DMFT) were used to evaluate the oral health status in the students with ASD in Tehran, Iran. A better oral hygiene status was related to higher brushing frequency and lower sweet snack consumption. The findings of the present study indicate that educational interventions regarding oral hygiene and healthy diet may improve oral self-care in individuals with ASD.

## Background

The term "autism" refers to autism spectrum disorders (ASD), a set of developmental, psychological, and neurological disorders that occur in early childhood [1]. The exact etiology of ASD is largely unknown. The most important characteristic of people with ASD is difficulty in social interactions and very poor communication, as well as behaviors, interests or activities that may be restricted and repetitive or have a stereotypical pattern. Autistic children often show stubbornness and endurance including focus on unusual activities, inflexible attachment to daily routine, resistance to any change, repetitive and stereotypical movements and activities, and unconventional attention to the details of objects [2].

The chance of autism is four times higher in boys compared to girls [3] and the disorder has been observed with varying severities across all races and groups around the world. Moreover, an increasing prevalence of autism is generally reported in the world. The prevalence of ASD is significant; for example, it was 25 per 1000 children aged 3–17 years in the USA in 2016 [4]. The incidence of autism was 1 in 110 American children or 1 in 70 American boys in 2007 [5]. In 2008, the incidence of autism was found to be 1.9% in Iran [6]. The prevalence of ASD was estimated at 6.6 per 10,000 children in Iran in 2012 [7].

Researchers in the field of oral health have not paid enough attention to these individuals with ASD due to specific autistic behavioral characteristics. Most of the studies have revealed that the oral health indices of these individuals are worse than the general population. There was no significant difference in the incidence of dental caries or the presence of malocclusions and oral habits between children with ASD and the healthy pediatric population. However, a poorer oral hygiene and gingival/periodontal state have been reported in these children compared to healthy children [8, 9]. A major characteristic of ASD patients is rigid, inflexible adherence to routines. The severity and extent of oral diseases and the resulting changes in the patient’s environment may provoke an aggressive outburst or tantrum [10]. This is while dental care is the most common unmet need in autistic individuals [8]. Some disorders such as reduced saliva due to prescription drugs, unhealthy eating habits, poor oral hygiene and harmful oral habits like bruxism can increase the risk of dental caries and periodontal diseases in children with autism. Poor oral health can lead to difficulty eating and speaking, mouth pain, sleep disorders, and low self-esteem, resulting in a negative impact on the person's health and quality of life [8, 11]. In addition, patients with ASD usually need to receive extensive dental services under anesthesia, which places a significant financial burden on the family and the health system [9]. To prepare autistic individuals for exposure to a new social situation such as clinical examinations, social learning tools including social stories are widely used. These stories are short by design and rely on a ratio of descriptive, perspective and/or affirmative sentences in addition to visual cues and make the individual familiar with the steps of the situations in advance [7].

The results of the studies regarding dental caries in patients with ASD are contradictory; however, they indicate the difference in the oral health status of the patients in developed and developing countries. Some studies, mostly those conducted in developed countries that have special care and training centers for children with autism such as the United States, Sweden and Japan, have reported a low prevalence of dental caries in these individuals [12–14]. On the contrary, in developing countries such as Pakistan, Egypt and the United Arab Emirates, most of the studies found a higher prevalence of dental caries in patients with autism compared to healthy children [15–17]. No national oral health survey has been conducted in the Iranian ASD population. However, in 2019, a case–control study in Rasht, one of the northern cities of Iran, demonstrated a poorer oral hygiene and gingival state along with a higher prevalence of dental caries in ASD children in comparison with healthy ones [18].

It seems necessary to conduct a study to gather the basic oral health information of Iranian autistic children due to the increasing prevalence of ASD and the importance of oral health in these individuals considering the gap of knowledge in this field in Iran. Therefore, a study was conducted to evaluate the oral health status of primary school students with ASD (7 to 15 years old) in autism schools in Tehran, Iran.

## Methods

### Study design and population

A descriptive/analytical cross-sectional survey was conducted in Tehran, Iran. All seven governmental special primary schools in the city of Tehran were selected for this study. None of the schools was a boarding school. Of 352 students with ASD aged 6–15 years old in the first to sixth grades, the parents of 265 students consented to their children's participation in the study by signing a written informed consent form after obtaining information about the purpose and process of the study via a letter sent to them along with the consent form by teachers (response rate: 75.2%). The inclusion criteria were parents’ consent, age between 7 and 15 years old, and an ASD level of 1 or 2 [19]. The exclusion criteria were the presence of other physical or developmental disabilities and lack of cooperation. All of the students were previously diagnosed with ASD by competent specialists of Tehran Exceptional Education Office and their medical records were made available to the authors.

A psychologist assessed the severity of autism (ASD level) according to the Diagnostic and Statistical Manual of Mental Disorders, 5th Edition (DSM-5) [19]. These levels included level one (mild ASD), level two (moderate ASD), and level Three (severe ASD). Students assigned to ASD level three were excluded from the study. Finally, 217 students were included in the study.

### The instrument

An instrument was designed to measure oral health behaviors and barriers to tooth brushing in autistic students. The questionnaire items were derived from relevant articles and resources and then reviewed and revised to prepare an initial draft of the instrument. An expert panel including eight oral public health specialists, an epidemiologist, and a health education specialist assessed the content validity of the questionnaire in terms of relevancy, clarity, simplicity, and necessity. Content validity index (CVI) and content validity ratio (CVR) were calculated for each item. All of the items had acceptable CVI (≥ 0.83) and CVR value (≥ 0.75) [20]. To evaluate the face validity of the questionnaire, it was completed by 12 parents of autistic children. The respondents were asked about the clarity and comprehensibility of the items, and it was found they considered the questionnaire acceptable in these aspects. The reliability of the instrument was tested from two aspects of internal reliability and stability. Cronbach's alpha coefficient was measured and test–retest was done by twelve participants at an interval of two weeks, which were both within acceptable limits (Cronbach's alpha coefficient = 0.96, and ICC > 0.70) [21].

The questionnaire consisted of three parts. The first part was about demographic and basic characteristics of the students including age, sex, parents’ educational level, and household economic status. The second part consisted of four multiple choice questions to evaluate oral health behavior of autistic children (including 1- tooth brushing frequency, 2- routines of tooth brushing-brushing with/without help-, 3- using fluoride containing tooth paste, and 4- frequency of daily Sugary snack). The third part contained seven yes/no questions indicating which one of the seven possible barriers and challenges the student faced with while brushing their teeth. These items included 1- tooth brushing as an effort and a time-consuming task, 2- tooth brushing as a complicated task, 3- dislike oral entry, 4- dislike tooth brushing as a task, 5- unwanted movements appear while doing the task. 6-fear of tooth brushing. 7- difficulty in tooth brushing.

### Pilot study and calibration

A pilot study was conducted in a private charity educational center for autistic children in Tehran. Eleven children with ASD who were not included in the main study participated in the pilot study. The inter-examiner agreement was assessed between the examiner and an oral public health specialist. First, the two examiners reviewed the examination method. They examined one child with ASD to observe the examination of each other and discuss coding issues. Accordingly, the 10 remaining children with ASD were examined by both examiners separately in the same room. (ICC = 1 for DI, CI and OHI-S and ICC = 0.83 for DMFT). For intra-examiner calibration, the examiner- a general dentist- examined 10 children to evaluate the Simplified Oral Hygiene Index (OHI-S) and DMFT (Decayed, Missing and Filled Teeth). The second assessment was after half an hour. (ICC = 1 for debris index (DI), calculus index (CI), OHI-S and DMFT).

### Data collection

Written informed consent was obtained from the parents. Subsequently, the questionnaire was completed by one of the parents and oral examination was done by the examiner. A week before the examination, a social story containing the pictures of the examiner and the steps of examination was given to the parents and they were asked to tell the story and show pictures to their children at least three times a day during the week.

On the examination day, the students entered the school health and care room one by one, accompanied by their teacher. Each student was requested to sit on the chair with help of the teacher. Before oral examination, two pictures were shown to the student, a picture of the examiner holding an examination mirror followed by a picture of a child undergoing oral examination by the examiner. The oral examination was carried out by the examiner sitting in front of the student. The examination was done under natural light using an intra-oral examination mirror. To assess the oral hygiene status, the OHI-S was recorded [22] including DI and CI. Accordingly, six index teeth [all 4 first molars [4], upper right and lower left central incisors [2] were considered and labial surfaces were examined except for the lower molars where the lingual surfaces were examined. Codes for DI were as follows: 0 = absence of soft debris or extrinsic stain, 1 = debris covering not more than one third of the tooth surface, 2 = debris covering more than one third but not more than two third of the tooth surface regardless of the presence of extrinsic stain, and 3 = soft debris covering more than two thirds of the examined tooth surface. The CI was scored as follows: 0 = no calculus present, 1 = supragingival calculus covering not more than a third of the exposed tooth surface, 2 = supragingival calculus covering more than one third but not more than two thirds of the exposed tooth surfaces or the presence of individual flecks of subgingival calculus around the cervical portion of the tooth or both, 3 = supragingival calculus covering more than two thirds of the exposed tooth surface or a continuous heavy band of subgingival calculus around the cervical portion of the tooth or both. In addition, Decayed, Missing and Filled Teeth in permanent dentition (DMFT), Decayed Teeth in permanent dentition (DT), Missing Teeth in permanent dentition (MT), and Filled Teeth in permanent dentition (FT) were recorded for each student. The cooperation level of the student with the dentist during examination was measured using the Frankle Index with a score range of 1–4 including 1: Definitely negative for refusal of examination, crying forcefully, fearful, or any other overt evidence of extreme negativism, 2: Negative for reluctant to accept examination, uncooperative, some evidence of negative attitude but not pronounced, sullen, withdrawn, 3: Positive for acceptance of the examination, at times cautious, willingness to comply with the dentist, but patient follows the dentist's directions cooperatively, and 4: Definitely positive for good rapport with the dentist, interested in the dental procedures, laughing and enjoying the situation [23]. A soft tooth brush was given to each student as a prize at the end of the oral examination.

Economic status was assessed according to relative poverty in urban areas of Iran, estimating that 360 USD provides the minimum subsistence needs for an urban family in Iran for a month. In this study, we asked the parents if their income was above or below this amount. An income of less than 360 USD per month was considered as low income and above 360 USD was considered as high income.

The education level of parents was assessed using their last academic certificate via a multiple-choice question ordinated in 6 levels of education including: 1- illiterate, 2- unfinished high school education, 3- high school diploma, 4- associated degree, 5- bachelor’s degree, 6- master’s degree or higher. Associated degree is an equivalent for a college education. Then, these levels were dichotomized according to our statistical distribution into two groups of associate degree or lower and above associate degree.

### Statistical analysis

The data was analyzed using SPSS version 24 (IBM, Armonk, NY, USA). First, the whole data was scanned and cleared from false entries. Then, missing values were imputed by Expectation Maximization (EM) [24]. Descriptive statistics of demographic and basic characteristics of the students with ASD, oral health behavior, tooth brushing barriers and the results of oral examinations were produced. The distribution of the quantitative variables is presented as mean and standard deviation. Qualitative variables are reported as percentage and number.

We classified the participants into two groups according to their age. The first group was in the range of 7 to 11 years and the second group was in the range of 12 to 15 years old. This classification was done as the number of the participants in each group was almost equal. Additionally, the first age group had mix dentition while the second group had permanent dentition.

The oral hygiene status assessed by the OHI-S was categorized into two groups for data distribution analysis according to a previous study [25] (OHI-S ≤ 1.83 as good oral hygiene status and OHI-S > 1.83 as poor oral hygiene). The effect of different sociodemographic and oral health behavior factors on the OHI-S in students with ASD was assessed using logistic regression analysis. The relationship between different factors and DMFT in all students with ASD was assessed using generalized linear model regression (negative binomial identity link function). *P*-values less than 0.05 were considered significant.

### Ethics

Ethical clearance was obtained from the Research Ethics Committee of the School of Dentistry, Tehran University of Medical Sciences. Written informed consent was obtained from parents before the study and they were assured that their identity remained anonymous (IR.TUMS.DENTISTRY.REC.1398.153).

## Results

A total of 217 students with ASD aged 7–15 years old participated in the study. Table 1 demonstrates the demographic and basic characteristics of the participants (Table 1) The oral hygiene status and caries experience of children with ASD were measured (mean DMFT = 2.36; SD, ± 2.38) and (mean OHI-S = 1.92; SD, ± 0.55) (Table 2). The oral health behaviors including oral hygiene and dietary habits in children with ASD are presented in Table 3.

**Table 1:** Demographic characteristics and basic information of children with ASD (total = 217)

	**7–11 years old** **(*n* = 109)**	**12–15 years old** **(*n* = 108)**	**Total** **(*n* = 217)**
**Age (years)**	9.40 ± 1.17	13.51 ± 1.18	11.45 ± 2.37
**Gender (boy):**	94 (86.2%)	92(85.2%)	186(85.7%)
**Father’s education level**			
Associate degree or lower	58(53.2%)	49(45.4%)	107(49.3%)
Above associate degree	51(46.8%)	59(54.6%)	110(50.7%)
**Mother’s education level**			
Associate degree or lower	58(53.2%)	54(50.0%)	112(51.6%)
Above associate degree	51(46.8%)	54(50.0%)	105(48.4%)
**Family income**			
Less than 360 USD	85(78.0%)	77(71.3%)	162(74.7%)
360 USD or more	24(22.0%)	31(28.7%)	55(25.3%)
**Level of ASD**			
1 (mild)	64(58.7%)	63(58.3%)	127(58.5%)
2 (moderate)	45(41.3%)	45(41.7%)	90(41.5%)
**Cooperation level (Frankle)**			
Definitely positive	44(40.4%)	56(51.9%)	100(46.1%)
Positive	34(31.2%)	33(30.6%)	67(30.9%)
Negative	24(22.0%)	16(14.8%)	40(18.4%)
Definitely negative	7(6.4%)	3(2.8%)	10(4.6%)

**Table 2:** Description of oral hygiene status and caries experience of children with ASD (total = 217)

	**7–11 years old** **(*n* = 109)**	**12–15 years old** **(*n* = 108)**	**Total** **(*n* = 217)**
**Clinical index**			
DMFT	1.96 ± 1.72	2.79 ± 2.85	2.36 ± 2.38
DT	1.50 ± 1.42	1.87 ± 2.01	1.70 ± 1.74
MT	0.05 ± 0.4	0.00 ± 0.00	0.02 ± 0.28
FT	0.42 ± 1.16	0.90 ± 2.10	0.66 ± 1.70
DI	1.92 ± 0.43	1.80 ± 0.46	1.86 ± 0.45
CI	0.05 ± 0.26	0.07 ± 0.20	0.06 ± 0.23
OHI-S	2.00 ± 0.55	1.90 ± 0.54	1.92 ± 0.55

**Table 3:** Description of oral health behaviors including oral hygiene and dietary habits in children with ASD (total number = 217)

	**7–11 years old** **(*n* = 109)**	**12–15 years old** **(*n* = 108)**	**Total** **(*n* = 217)**
**Brushing frequency**			
More than once a day	9(8.3%)	13(12.0%)	22(10.1%)
Once a day	55(50.5%)	65(60.2%)	120(55.3%)
Two or three times a week	25(22.9%)	17(15.7%)	42(19.4%)
Once a week	7(6.4%)	4(3.7%)	11(5.1%)
Never or irregular	13(11.9%)	9(8.3%)	22(10.1%)
**Daily Brushing frequency (dichotomous)**			
Once or more	64(58.7%)	78(72.2%)	142(65.4%)
Less than once	45(41.3%)	30(27.8%)	75 (34.6%)
**Tooth brushing routine**			
Alone without supervision	7(6.4%)	19(17.6%)	26(12.0%)
Alone with supervision	20(18.3%)	26(24.1%)	46(21.2%)
With the help of caregivers	34(31.2%)	35(32.4%)	69(31.8%)
Only by caregivers	40(36.7%)	24(22.2%)	64(29.5%)
Never brushes	8(7.3%)	4(3.7%)	12(5.5%)
**Tooth brushing routine (dichotomous)**			
Unaided	7(6.4%)	19(17.6%)	26(12.0%)
With help	102(93.6%)	89(82.4%)	191(88.0%)
**Using fluoride containing tooth paste**			
Always	41(37.6%)	56(51.9%)	97(44.7%)
Most of the time	31(28.4%)	32(29.6%)	63(29.0%)
Seldom	15(13.8%)	13(12.0%)	28(12.9%)
Never	15(13.8%)	3(2.8%)	18(8.3%)
No idea	7(6.4%)	4(3.7%)	11(5.1%)
**Using fluoride containing tooth paste (dichotomous)**			
Most of the time	72(66.1%)	88(81.5%)	160(73.7%)
Seldom or never	37(33.9%)	20(18.5%)	57(26.3%)
**Frequency sugary snack consumption per day**			
Seldom or never	9(8.3%)	6(5.6%)	15(6.9%)
Sometimes	32(29.4%)	29(26.9%)	61(28.1%)
Almost once	21(19.3%)	27(25.0%)	48(22.1%)
Almost twice	30(27.5%)	32(29.6%)	62(28.6%)
Three times or more	17(15.6%)	14(13.0%)	31(14.3%)
**Frequency of sugary snack consumption per day (dichotomous)**			
Twice or less	92(84.4%)	94(87.0%)	186(85.7%)
More than twice	17(15.6%)	14(13.0%)	57(26.3%)

**Fig.1:**
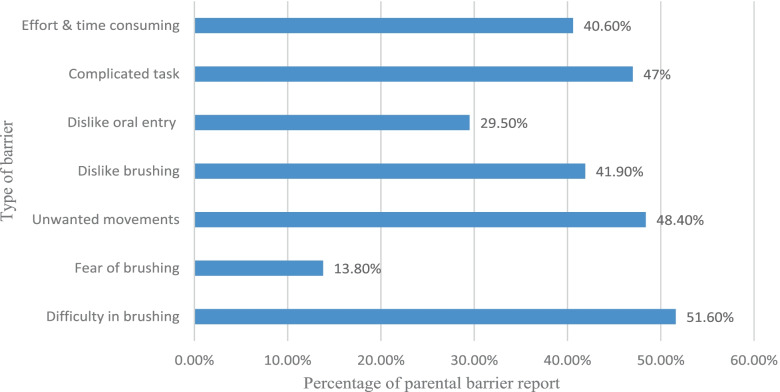
Percentage of tooth brushing barriers reported by parents of children with ASD (*n* = 217)

The percentages of the barriers to tooth brushing reported by the parents were calculated. The most reported barrier to tooth brushing in children with ASD was “difficulty in brushing = 51.6%” and the least reported barrier was “student’s fear of brushing = 13.8%” (Fig. 1).

The relationship between different sociodemographic and oral health behavior factors and the oral hygiene status (OHI-S) in all autistic students and their subgroups according to brushing frequency as an effect modifier in relation to OHI-S was evaluated (Table 4). Unadjusted data analysis of the correlation of each independent factor with the oral hygiene status in all students (*n* = 217) showed that poor oral hygiene status (OHI-S score above 1.83) was significantly higher in the age group 7–11 years compared to those aged 12–15 years, in students that brushed their teeth less than once a day versus those who brushed once or more daily, in students who brushed with the help of a caregiver versus those who brushed unaided, and in students who had sweet snacks more than twice a day compared to those who consumed sugary snacks less than twice a day (Table 4). Multivariable regression analysis of the relationship between oral hygiene status and different factors showed that the only significant factor was age (OR = 1.90, 95% CI: 1.09, 3.31; *p*-Value = 0.02). After removing age, the association of tooth brushing frequency (OR = 1.80, 95% CI: 1.02, 3.18; *p*-Value = 0.04) and sweet snack consumption (OR = 2.23, 95% CI: 1.00, 3.31; *p*-Value = 0.05) with OHI-S remained significant.

**Table 4:** Correlation of different sociodemographic and oral health behavior factors with oral hygiene status in all autistic children and their subgroups according to brushing frequency as an effect modifier in relation to OHI-S

	**Students who brush once or more daily (*n* = 142)**		**Students who brush less than once daily (*n* = 75)**		**Total students (*N* = 217)**	
	Unadjusted OR (95%CI)	*p*-Value^a^	Unadjusted OR (95%CI)	*p*-Value^a^	Unadjusted OR (95%CI)	*p*-Value^a^
**Age**						
7–11 years	2.73 (1.37, 5.41)	0.001	1.05 (0.41, 2.66)	0.92	2.07 (1.21, 3.56)	0.008
12–15 years	1		1		1	
**Gender**						
Boy	0.64 (0.26, 1.57)	0.33	0.41 (0.08, 2.19)	0.30	0.62 (0.29, 1.34)	0.23
Girl	1		1		1	
**Family income**						
$360 or more	1		1		1	
Less than $360	0.55 (0.26,1.16)	0.12	0.81 (0.28, 2.40)	0.71	0.64 (0.34, 1.18)	0.15
**Level of ASD**						
1(mild)	1.12 (0.57, 2.20)	0.75	1.08 (0.43, 2.72)	0.87	1.09 (0.64, 1.87)	0.76
2(moderate)	1		1		1	
**Father’s education level**						
Associate degree or lower	1.10 (0.56, 2.13)	0.79	1.15 (0.46, 2.88)	0.77	1.14 (0.67, 1.94)	0.64
Above associate degree	1		1		1	
**Mother’s education level**						
Associate degree or less	0.76 (0.39, 1.49)	0.42	0.73 (0.28, 1.88)	0.51	0.82 (0.48, 1.40)	0.47
More than Associate degree	1		1		1	
**Daily brushing frequency**						
Once or more	-	-	-	-	1	
Less than once	-	-	-	-	1.78 (1.01, 3.14)	0.045
**Tooth brushing routine**						
Alone Without help	1		1		1	
With help	7.83 (1.74, 35.37)	0.01	0.51 (0.10, 2.80)	0.44	2.8 (1.13, 6.97)	0.03
**Using fluoride containing tooth paste**						
Most of the time	1		1		1	
Seldom	0.86 (0.35, 2.08)	0.74	1.16 (0.46, 2.93)	0.76	1.17 (0.64, 2.15)	0.60
**Frequency of sugary snack consumption per day**						
Once or twice	1		1		1	
More than twice	2.86 (1.07, 7.70)	0.04	1.36 (0.36, 5.12)	0.65	2.21 (1.00, 4.87)	0.049

^a^Simple Logistic Regression analysis

Tooth brushing frequency was found to be an effect modifier variable of the oral hygiene status (OHI-S) as the outcome. Therefore, the findings were analyzed separately according to brushing frequency. The results of unadjusted data analysis are presented in Table 4. Multivariable analyses in the subgroup of students who brushed once daily or more (*n* = 142) showed that a poor oral hygiene status was significantly higher in the age group 7–11 years versus those aged 12–15 years (OR = 2.46, 95% CI: 1.20, 5.04; *p*-Value = 0.01), in those who brushed with the help of a caregiver compared to who brushed unaided (OR = 6.74,95% CI: 1.46,31.12; *p*-Value = 0.01), and in the students who had sweet snacks more than twice a day versus those who had sweet snacks less than twice a day (OR = 2.90, 95% CI: 1.00, 8.40; *p*-Value = 0.05). However, no statistically significant relationship was observed between OHI-and any of the factors in the subgroup of students who brushed less than once a day (*n* = 75).

The relationship of different sociodemographic and oral health behavior factors with DMFT was evaluated in the autistic children. The results of unadjusted data analysis are demonstrated in Table 5 in detail. It was found that age and oral hygiene status had a significant relationship with DMFT, and DMFT increased with an increase in age and the OHI-S (Table 5). In multivariable analysis, the correlation of age (Regression coefficient = -0.84, 95% CI: -1.6, -0.1; *p*-Value = 0.03) and OHI-S (Regression coefficient = -0.8, 95% CI: -1.5, -0.1; *p*-Value = 0.03) with DMFT remained statistically significant.

**Table 5:** Correlation of different sociodemographic and oral health behavior factors with DMFT in all autistic children

	**DMFT**	**Regression coefficient**	**CI:95%**	***p*-Value**^**a**^
**Age**				
7–11 years old (*n* = 109)	1.96 ± 1.72	-0.81	-1.5, -0.14	0.04
12–15 years old (*n* = 108)	2.79 ± 2.85	Reference		
**Gender**				
Boy (*n* = 186)	2.31 ± 2.31	-0.40	-1.59, 0.78	0.51
Girl (*n* = 31)	2.71 ± 2.81	Reference		
**Family income**				
360 USD or more (*n* = 162)	2.23 ± 2.31	Reference		
Less than 360 USD (*n* = 55)	2.75 ± 2.56	-0.51	-1.45, 0.43	0.29
**Level of ASD**				
1(mild) (*n* = 127)	2.26 ± 2.43	-0.25	-1.03, 0.52	0.53
2(moderate) (*n* = 90)	2.51 ± 2.31	Reference		
**Father’s education level**				
Associate degree or lower (*n* = 107)	2.50 ± 2.46	0.26	-0.50, 1.01	0.50
Above associate degree (*n* = 110)	2.24 ± 2.31	Reference		
**Mother’s education level**				
Associate degree or lower (*n* = 112)	2.18 ± 2.23	-0.38	-1.14, 0.38	0.32
Above associate degree(*n* = 105)	2.56 ± 2.53	Reference		
**Daily brushing frequency**				
Once or more Regularly(*n* = 142)	2.37 ± 2.51	0.006	-0.78, 0.80	0.99
Irregularly or no brushing(*n* = 75)	2.36 ± 2.14	Reference		
**Tooth brushing with or without help**				
Alone Without help (*n* = 26)	2.31 ± 3.10	-0.06	-1.20, 1.07	0.91
With help(*n* = 191)	2.37 ± 2.28	Reference		
**Using fluoride containing tooth paste**				
Most of the time(*n* = 160)	2.34 ± 2.24	-0.07	-0.94, 0.79	0.86
Seldom (*n* = 57)	2.42 ± 2.76	Reference		
**Frequency of Sugary snack consumption per day**				
Once or twice (*n* = 186)	2.29 ± 2.43	-0.52	-1.73, 0.70	0.41
More than twice(*n* = 31)	2.81 ± 2.04	Reference		
**OHI-s**				
= < 1.83 (*n* = 113)	1.89 ± 2.41	-0.76	-1.5, -0.03	0.04
> 1.83 (*n* = 104)	2.65 ± 2.32	Reference		

^a^Generalized linear model regression (negative binomial identity link function)

## Discussion

This cross-sectional study provided an overview of dental caries experience and oral hygiene status in students with ASD in primary schools (aged 7–15 years old) as well as their cooperation level during oral examination. In addition, oral health behaviors and barriers to tooth brushing were evaluated in the participants.

Previous studies in Iran have been reported the mean DMFT of individuals without ASD which seemed considerably lower than the mean DMFT of autistic students in the present study [26–28].

The results of previous studies regarding dental caries experiences in patients with ASD in different countries are controversial. Most Studies in developed and newly industrialized countries [29], where there are special care and training centers for individuals with ASD, have reported low caries experience in the ASD population. These studies contained the United States [13], China [30], India [31], Turkey [32] Sweden [14] and Japan which found that the experience and severity of dental caries showed a marked decrease from previous observations in children with ASD. The results showed that the social context of dental treatment for physically challenged children improved during the past 15 years. Clinic attendance showed a clear progression leading to the improvement of compliance with routine dental checkups. Increased frequency of tooth brushing and fewer sugary snacks eaten by patients between meals were also observed. These results suggested a possible improvement in the attitude toward dental health and the caregiver’s behavior aiming at dental caries prevention [33]. In the present study, we provided the clinical and behavioral oral health information of children with ASD, which could be a basis for future longitudinal studies.

On the contrary, several studies reported a higher prevalence of dental caries in individuals with ASD compared to general population in some developing countries, where people often perceive the quality of health care and education as poor [34]. Such studies contained Yemen [35], Pakistan [17], the United Arab Emirates [16], and Iran which was almost in line with the findings of the present study [18].

However, a meta-analysis found no significant difference in DMFT/dmft between the two groups of individuals with and without ASD in the world. In the Asian population, a significantly higher dmft index has been reported in children with ASD compared to the controls, but such a difference has not been found in the non-Asian population. Although the reason remains largely unknown, this variation may be partially explained by differences between western and eastern cultures in terms of dietary habits and patterns of dental visits [36]. Moreover, a recent systematic review showed no significant difference in the caries prevalence and severity between those with and without ASD. A systematic review evaluated caries prevalence in six studies [14–16, 30, 32, 37] of which two studies reported a significantly lower caries prevalence among individuals with ASD, one study found the opposite [16] and the rest of the studies found no difference between the two groups [14, 15, 37]. Nevertheless, in conclusion more high-quality case–control studies were warranted to provide a more descriptive and precise picture of the oral health status of ASD individuals [38].

The findings showed that the mean (DT + dt) was considerably higher than the mean (FT + ft) in all students with ASD, which was consistent with other studies [16, 18]. These results could be due to the poor dental knowledge and poor cooperation of these children with dentists or parents being too exhausted to have constant supervision over their children to follow oral hygiene instructions [18]. In addition, inadequate training of dentists, dental specialists, and hygienists and high sensitivity of these children to unfamiliar sounds, lights, odors and colors, are barriers to accessing dental care [39].

In the present study, students with ASD had poor oral hygiene. This finding was consistent with previous studies [8, 15, 16, 31, 40–44]. However, a recent systematic review found no difference in the oral hygiene status between those with and without ASD [38].

The cooperation level of students with ASD during oral examination was mostly rated as positive and definitely positive by the examiner in the present study, which was in line with the results of one study [15] but in contrast to some other studies [18, 31, 45]. This could be not only because of the exclusion criteria of the present study excluding students with severe ASD, but also due to the absence of autistic children who could not pass the entrance exam of governmental schools and were homeschooled or attended private centers; these children usually have severe ASD or worse educational and behavioral conditions.

In our study, about two-thirds of the students with ASD brushed their teeth once a day or more, which was in line with the findings of some studies [15, 17, 18, 37, 39, 46] but in contrasts to the results of one study [45]. Most of the participants brushed their teeth with help of a caregiver, which was also consistent with some other studies [15, 17, 37, 39, 45, 46]. More than two-thirds of the students used fluoride containing tooth paste most of the time, which was in agreement with previous studies [15, 17, 39, 46]. The majority of the participants had sugary snacks twice a day or less often, which was in line with previous studies as well [15, 17, 18, 32, 39]. However, an Indian case–control study found no significant difference in sugar exposure of the children between the ASD and healthy control group although ASD children having significantly higher number of eating events per day. The amount of cariogenic sugar in their daily diet and rewarding foods seems to play an important role in their sugar exposer [47]. Therefore, It is recommended that parents be educated on maintaining good oral hygiene for autistic children, which is an integral part of the optimal dental health via proper diet, dietary habits, and adequate oral hygiene [48].

Preventive strategies are needed for hypersensitivities and the sensory processing difficulties of autistic children. “Sensory over-responders” might face oral hygiene barriers; for instance, they may dislike the taste and texture of some types of toothpaste and gag with toothbrushing [49]. In the present study, among seven possible barriers to tooth brushing in the students with ASD, the most common barriers found were “difficulty in brushing” and “Unwanted movements while brushing” in line with previous studies [39, 47]. It is essential to discover and better understand the challenges and barriers to oral self-care in children with ASD.

In the present study, neither the economic status nor the education level of the parents was associated with the oral hygiene status or caries experience, which was in contrast to the results of some studies [50, 51]. It may be because of the fact that some families in the high socioeconomic class of the society prefer to send their autist children to some special private educational centers and schools. Moreover, some children are privately tutored. Thus, they were not available for this study.

No association was found between caries experience and oral hygiene status with the gender of the autistic students, which was in line with previous studies [18, 43] It could be due to the smaller number of girls in comparison with boys in this study.

Caries experience was related to oral hygiene status in the present study, which was consistent with the findings of other studies that found a poor oral hygiene was positively associated with more caries experience [8, 52]. Therefore, an oral health program that emphasizes prevention is considered of particular importance for children and young people with autism [16].

The Oral Hygiene Status was better in the age group 12–15 years old compared to the age group 7–11 years, which could be due to the development of self-care skills over time such as dental plaque removal by tooth brushing, flossing, etc.

It is noteworthy that the healthy students in the first to sixth grade of primary school are 7–12 years of age according to the Iran’s national educational program. However, autistic students may start their school a couple of years later than regular healthy students due to their developmental and learning limitations so that the age range in autism primary schools is wider than regular schools.

For further investigation, we controlled the effect of age. Iranian autistic children who had sugary snacks less than twice a day and brushed their teeth once a day or more had better oral hygiene status as expected.

The present study showed that among the autistic children who brushed at least once a day, the low aged ones and those who brushed with the help of a caregiver had poorer oral hygiene compared to those aged 12 to 15 years old and brushed unaided. These findings might indicate that children who had high living skills demonstrated a significantly better oral hygiene [37]. Furthermore, among these students, the oral hygiene was poor in those who had sugary snacks more than twice a day compared to those who had sweet snacks less than twice a day. However, among the students who brushed their teeth less than once a day, no difference was observed in the oral hygiene status regarding the age range, sweet snack consumption or routines of tooth brushing. This may be due to the strong effect of the frequency of tooth brushing and plaque removal on better oral hygiene status; thus, when this factor is markedly low, it may almost fade the effect of the other existing factors on promoting the oral hygiene status. The DMFT had a positive association with age and the OHI-S score, which was in line with previous studies that reported potential caries risk factors in individuals with ASD were predominantly related to oral hygiene status and age [8].

### Limitations

The students with ASD level 3 were excluded from this cross-sectional study due to the lack of cooperation during oral examination. Although there are special private centers and schools for children with ASD, we selected the study population from governmental schools since most of them had low to moderate severity levels of ASD and were relatively cooperative. In addition, significant comparisons between previous studies were limited because most of them were conducted in wider age ranges or in other countries with different conditions such as cross-cultural differences in living standards, dietary habits, genetics, etc.

### Strengths

Most of the studies on individuals with ASD are conducted in small populations or in wide age ranges. The present study evaluated an appropriate sample size of school students with ASD to assess their oral health status. Our study population included almost all of the students with ASD level 1 and 2 in Tehran, the capital of Iran and a multicultural city with a population of over 10 million with different socio-demographic characteristics, health beliefs, and races from all over the country. To the best of our knowledge, this descriptive study provides a great deal of basic information about oral health of children with ASD in Iran for the first time. Despite all limitations and high sensitivity of parents, educational authorities, and school staff, we managed to collect acceptable data for the study. Another advantage was the study setting. For autistic children, the school is the best place for examinations, since they are used to this environment. A change of location for dental examination would probably provoke negative behaviors in this group of children [5].

## Conclusion

The clinical indices of OHI-S and caries experiences (DMFT) were used to evaluate the oral health status of primary students with ASD in Tehran, Iran.

A better oral hygiene status was associated with a higher brushing frequency and a lower sweet snack consumption. This study provides researches with information on the oral health status in children and adolescents with ASD in Iran. The findings of this study suggest that preventive educational interventions with emphasis on healthy diet and teaching an easy, proper and effective tooth brushing technique may be desirable to improve oral self-care and improve the oral health, particularly in young individuals with ASD.

## Data Availability

The datasets used and analyzed during the current study contain information for a further clinical trial and are not publicly accessible at the time. However, they are available from the corresponding author on reasonable request.

## Abbreviations

OHI‑S: Oral Hygiene Index‑Simplified
ASD: Autism spectrum disorders
DMFT: Decayed, Missing, And Filled Teeth
CI: Calculus Index
DI: Debris Index
DT: Decayed Teeth
MT: Missing Teeth
FT: Filled Teeth
CVI: Content Validity Index
CVR: Content Validity Ratio
ICC: Intra-Class Correlation Coefficient
DSM-5: Diagnostic and Statistical Manual of Mental Disorders, 5th Edition
SPSS: Statistical Package for the Social Sciences
US: United States
EM: Expectation Maximization
95% CI: 95% Confidence Interval
OR: Odds Ratio
SD: Standard Deviation
